# Stereosynthesis of stationary phase with precision defined separation selectivity

**DOI:** 10.1039/d6sc05966j

**Published:** 2026-09-24

**Authors:** Xiangyu Huang, Kaiyue Sun, Hanchen Cao, Zhuoheng Zhou, Jikai Chen, Qingshu Cai, Xiaofei Wang, Yanling Song, Teng-Xiang Huang, Ning Fang, Bo Zhang

**Affiliations:** a Department of Chemistry and the MOE Key Laboratory of Spectrochemical Analysis & Instrumentation, College of Chemistry and Chemical Engineering, State Key Laboratory of Vaccines for Infectious Diseases, Xiang An Biomedicine Laboratory, Xiamen University Xiamen 361005 China bozhang@xmu.edu.cn; b Beijing Institute of Smart Energy Beijing 102211 China; c Bioanalytical Services, Department of Toxicology, WuXi AppTec (Suzhou) Co. Ltd Suzhou China; d ColumnScientific Inc. Xiamen 361102 China; e Pen-Tung Sah Institute of Micro-Nano Science and Technology, Xiamen University Xiamen 361005 China

## Abstract

Chemical separation is based on molecules' differential interactions with a stationary phase and a mobile phase. To enable efficient thermodynamics and molecular kinetics, micrometer-size spheres with bonded chemistry have become the general choice for stationary phase materials. For half a century, the application of stationary-phase chemistry to microspheres has been a random, unordered and bulk process in the manufacture of chromatographic materials. Such a non-stereoresolving synthetic strategy results in the co-location of multiple functional groups, which share the same space, including those that are designed to function with different or opposite selectivities, such as hydrophobic and hydrophilic, cation exchange and anion exchange, and so on. The co-location of these functional ligands can cause severe interference with each other, leading to deviation from, or even having a detrimental effect on, the designed separation selectivity. This non-spatially resolved stationary-phase chemistry diminishes the effectiveness of mixed-mode separation and constrains the feasibility and freedom of multi-functionality assemblies when pursuing high separation performance. In this study, through microfluidic precision manufacturing, microspherical stationary phases were stereosynthesized with well-defined functional domains and the realization of quantitatively tunable chemical selectivity. With a classical mixed-mode stationary phase as a benchmark, it was found that the originally existing, but seriously constrained, chromatographic retentivity can now be unleashed by the spatially resolved arrangement of chemical functionality. The current study suggests that, as an unexplored structural parameter, the spatial distribution of stationary phase chemistry is an independent characteristic, which can enable the precision design and free tuning of thermodynamics, providing a new approach towards high-performance chromatography.

## Abstract 2

The stereosynthesis of stationary phases defines functional domains at cubic-micron resolution and unleashes originally existing, but severely constrained, chromatographic thermodynamics, paving the way towards precisely defined chemical selectivity.

## Graphical abstract

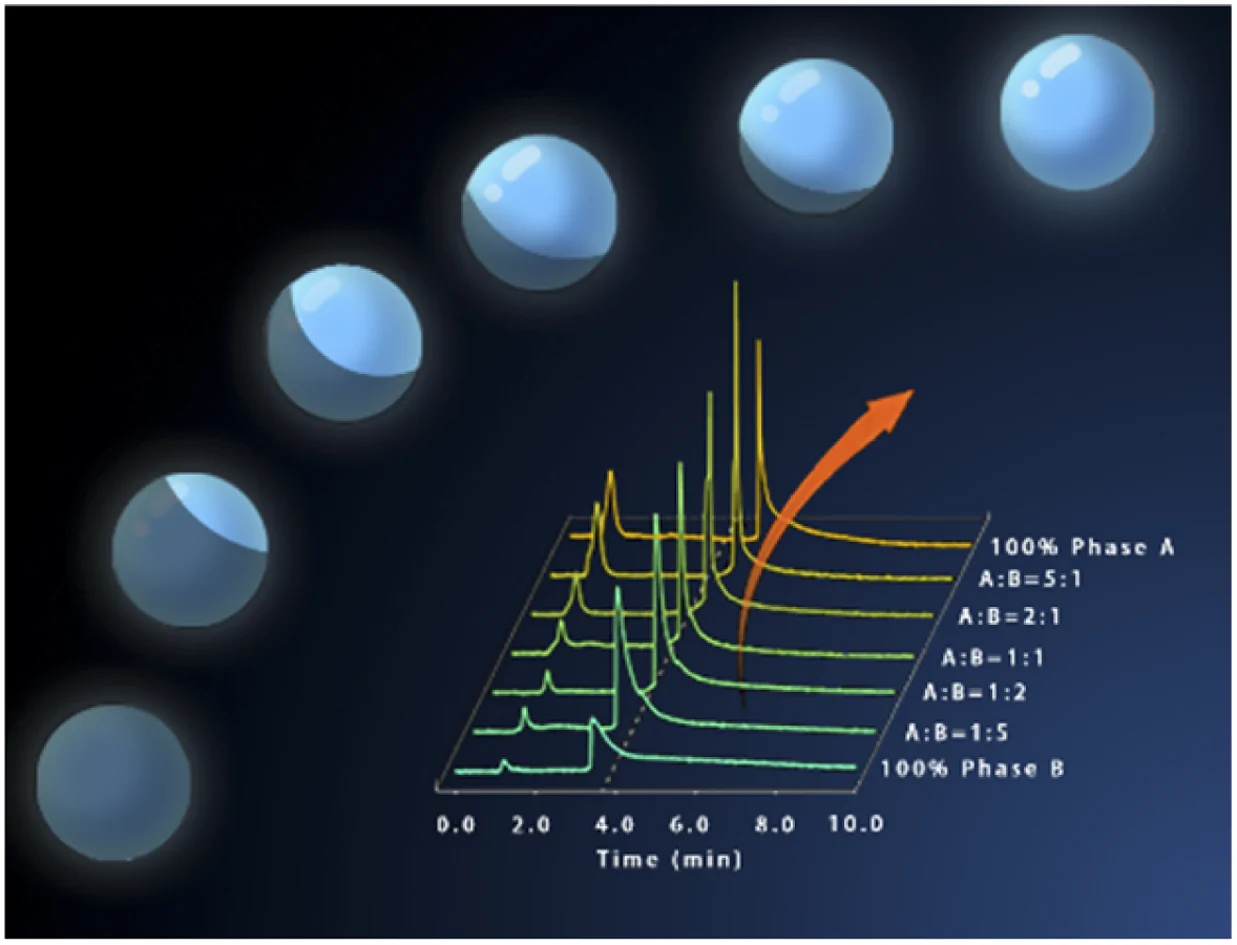

## Introduction

As one of the most powerful and widely used modern separation technologies, high-performance liquid chromatography (HPLC) has always sought materials with controllable retention behaviour and high separation selectivity for use with molecular mixtures.^1–3^ These thermodynamic features define the equilibrium process of solute molecules between the stationary phase and mobile phase, serving as the determinant of effective resolution.^4–6^ Although a number of stationary phase materials have been developed and commercialized, there remains a demand for broad and diverse selectivities to address emerging separation challenges where multiple interactions are involved.^7–11^ By combining a variety of selectivity mechanisms (*e.g.* hydrophobic, hydrophilic, ion exchange, *etc.*), the so-called mixed-mode stationary phases have demonstrated unique selectivity and enabled the resolution of molecules that are difficult to separate based on a single interaction.^12–15^

Currently, mixed-mode separation materials are typically prepared *via* batch processing strategies, *i.e.* multiple ligands bearing various functionalities are randomly bonded onto a microsphere surface without precise control over their quantity or spatial distribution.^4–6,16^ From the perspective of molecular interactions, however, such an uncontrolled and unclear combination of multiple functionalities may lead to non-ideal competition or intershielding of expected interactions, thereby affecting the realizable selectivity.^17,18^ As a result, even for stationary phases with identical chemical compositions, the effective selectivity may differ when the functional groups are applied with different spatial distributions.^19^ Unfortunately, the spatial distributions of stationary phase chemistry, on the scale of cubic micrometers, have long been disregarded and unexplored due to a lack of synthetic methodologies that could enable precise control of domain chemistry at such a scale. Since the inception of bonded-phase chromatography in the last century,^4^ the spatial distribution of stationary phase chemistry has rarely been treated as an independent consideration, resulting in uncontrollable and unpredictable effective separation selectivity being experienced by analyte molecules and therefore leading to passive and empirical, rather than rational and predictable, separation performance. Taking into account the quantitation and localization of functional groups as an essential dimension, there is a need to spatially resolve the architecture of the stationary phase through top-down design and stereosynthetic strategies.

In this work, we propose a precisely defined stationary phase based on micrometer-scale stereosynthesis (Scheme 1). Different chemical groups are introduced onto distinct areas of a microsphere in a Janus structure, enabling the independent and quantitative control of functionalities within a single micrometer-sized spherical entity. The stereosynthesis strategy enabled the preparation of monodisperse microspheres with sizes suitable for HPLC, and they demonstrated compatibility with a wide range of monomers, covering multiple types of stationary phase chemistry: hydrophobic, hydrophilic, cation exchange, anion exchange, *etc.* The spatially resolved stationary phases were shown to enable the accurate regulation of chemical selectivity and serve as model materials for understanding the influence of the spatial distribution of functional groups on chromatographic performance, offering a new perspective and approach for the rational design of multi-interaction chromatography and the precision manufacture of stationary phases with pre-defined selectivity.

**Scheme 1:**
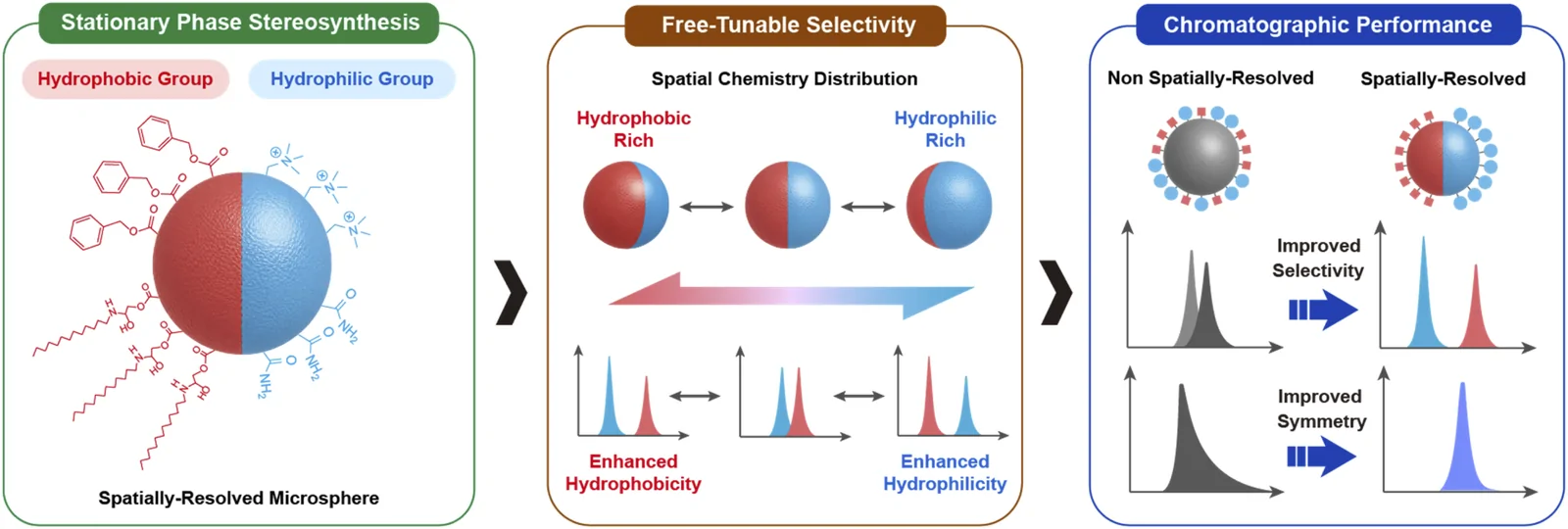
A schematic diagram of the modulation of a spatially resolved stationary phase with a quantitative spatial chemistry distribution, adjustable selectivity and enhanced chromatographic performance.

## Results and discussion

### Stereosynthesis of stationary phase microspheres

Spatially resolved stationary phases were synthesized using a microfluidic platform (Fig. 1), as we recently developed.^20–23^ Using Janus-structured monodisperse droplets generated by the microfluidic platform as templates, microspheres with a narrow particle-size distribution and consistently well-defined hemispherical resolution were synthesized. The microfluidic chip (Fig. 1a), with four identical channels, enables the high-throughput generation of droplets. Specifically, each channel for droplet generation consisted of two dispersed phases and a fluorinated oil continuous phase. Immiscible H_2_O and dimethyl carbonate (DMC) were used as solvents for the dispersed hydrophilic phase A and hydrophobic phase B, respectively, in which hydrophilic (for dispersed phase A) or lipophilic (for dispersed phase B) acrylate monomers and photoinitiators were dissolved as reaction precursors for free-radical polymerization. Monodisperse Janus droplets were emulsified in the fluorinated oil continuous phase (Fig. 1b), then solidified by ultraviolet (UV) irradiation and collected *via* a transparent Teflon tube (Fig. 1c). The prepared micrometer-scale droplets and microspheres exhibited good monodispersity (Fig. 1d–g) and clear phase interfaces (Fig. 1b and c), demonstrating the high definition of stereosynthesis.

**Fig. 1:**
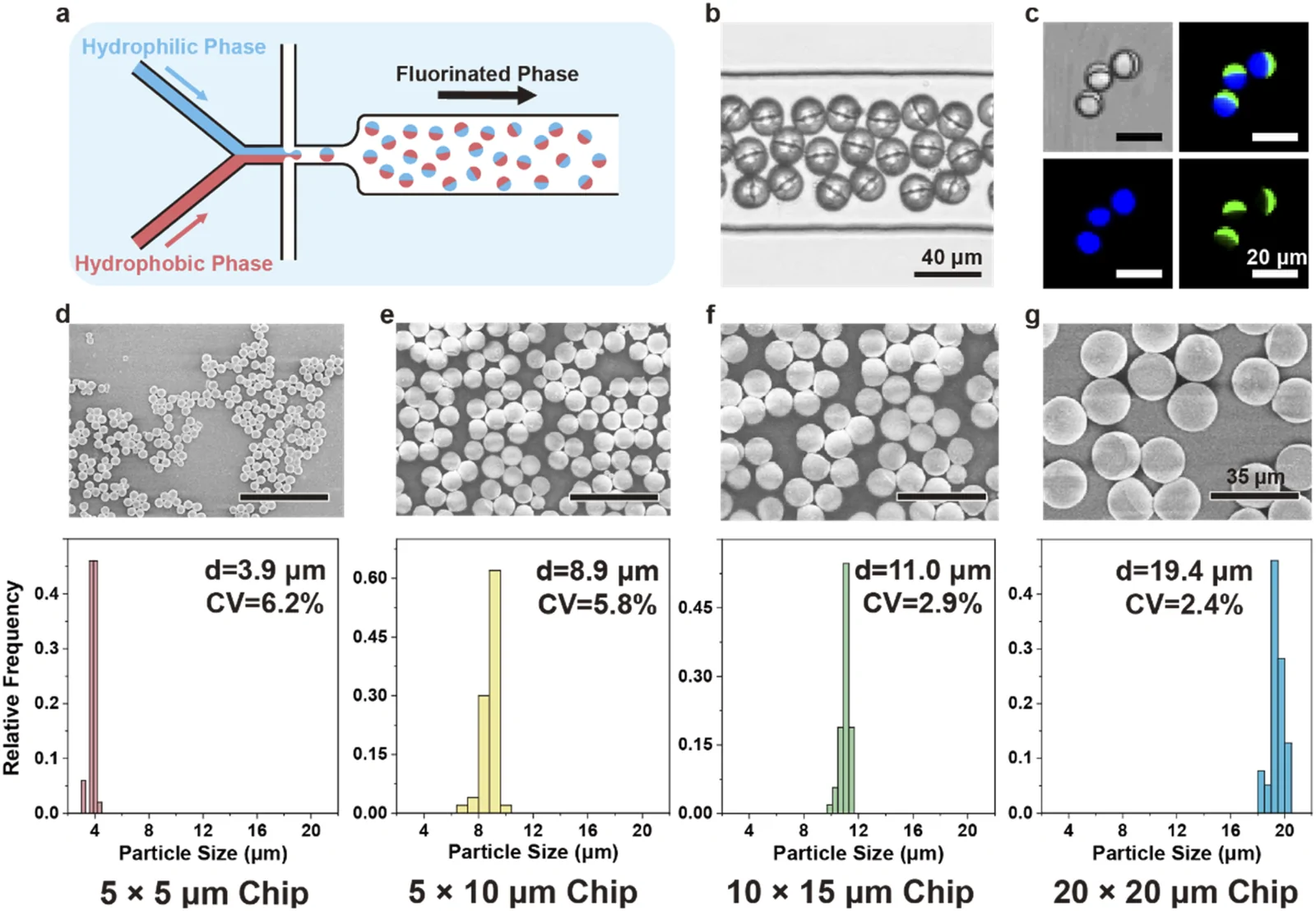
The stereosynthesis and characterization of spatially resolved stationary phases. (a) The microfluidic platform for the stereosynthesis of monodisperse spatially resolved stationary phase microspheres. (b) Optical microscopy imaging of the monodisperse spatially resolved droplets generated by the microfluidic device. (c) Fluorescence microscopy imaging of the spatially resolved microspheres formed by the solidification of droplets. (d–g) Spatially resolved microspheres exhibiting good monodispersity over a broad size range, which was achieved by adjusting the dimensions of the microfluidic channels.

The solvent and surfactant used in the continuous phase played a crucial role in the stable generation of spatially resolved droplets. Fluorinated oil (a mixture of hydrofluoroether and fluorocarbon, as detailed in Fig. S1), due to its immiscibility with both water and most organic solvents, is an excellent medium used for the continuous phase. The mutual immiscibility among H_2_O, DMC and fluorinated oil ensures the Janus structuring of the droplets and the resultant microspheres. Meanwhile, the low interfacial tension and low viscosity of fluorinated oil allow the stable generation of droplets within the microfluidic channels.^24–27^ Moreover, triblock copolymers (PFPE-PEG-PFPE and PFPE-PPG-PFPE), synthesized *via* a two-step reaction (Fig. S2), were used as fluorosurfactants to prevent droplet coalescence, maintaining a stable Janus emulsion during the microfluidic production process. The fluorosurfactants feature perfluoropolyether (PFPE) segments at both ends, with a hydrophilic polyethylene glycol (PEG) middle segment, or polypropylene glycol (PPG) in cases where the droplets contain highly lipophilic components (Fig. S3).

A key merit of microfluidic-based stereosynthesis is that, without disturbing the hemisphere structure, the particle size of the Janus stationary phase can be freely tuned by adjusting the dimensions of the microfluidic channels. Technically, although small numbers of Janus droplets/spheres with dimensions ranging from 10 s to 100 s of µm have been prepared before,^24,28,29^ the synthesis of Janus droplets/spheres with a size of 10 µm or smaller and at high throughput is still a challenge. It needs to be highlighted that, for modern liquid chromatography, only microspheres with a size ≤10 µm are capable of showing high separation performance.^30,31^ As shown in Fig. 1d–g, nonporous microspheres with sizes ranging from 3.9 to 19.4 µm were successfully synthesized using junctions ranging from 5 × 5 µm to 20 × 20 µm (height × width). Of these, the 10 µm microspheres were chosen for the following chromatographic studies. In all cases, the generated droplets exhibited a clearly defined Janus structure and maintained good monodispersity (CV ≤ 6.2%). The consistency of the four identical channels was also evaluated (Fig. S4). Owing to the equivalent design in terms of flow resistance, droplets generated among the multiple channels were consistent, and the microspheres collected from the four channels exhibited good uniformity (CV = 2.4–6.2%) at a high production throughput of 4000 Hz per chip. Within one hour, the generated microspheres can support the manufacture of four HPLC columns (150 µm i.d. × 100 mm length).

### Precision assembly of stationary phase chemistry

Based on the droplet microfluidic platform developed above, stationary phase chemistry can be applied with spatial resolution. Owing to the mutually immiscible synthetic system, a variety of monomers (acrylates or allyl derivatives, *e.g.* acrylamide, 3-((2-(methacryloyloxy)ethyl)dimethylammonio)propane-1-sulfonate (SBMA), 2-hydroxyethyl methacrylate (HEMA), allyltrimethylammonium chloride, benzyl methacrylate, 10-undecenoic acid, and glycidyl methacrylate (GMA)) can be infused into the two dispersed phases, respectively, thereby allowing the versatile assembly of functionalities onto the Janus microspheres. As shown in Fig. 2, various types of stationary phase chemistry have been successfully assembled, covering diverse chromatographic modes.^4–6^ These included alkyl and phenyl functionalities for reversed-phase chromatography (RPC), amide and hydroxyl functionalities for hydrophilic interaction chromatography (HILIC), and carboxyl and ammonium functionalities for ion-exchange chromatography (IEC), the three most efficient HPLC modes. It needs to be highlighted that epoxy groups can also be introduced into the lipophilic phase. These groups are typically used as ring-opening reaction sites, opening a wealth of chemical selectivities through the further introduction of various functional groups. In this study, the dodecyl group was introduced *via* the ring-opening reaction of the epoxy group with dodecylamine.

**Fig. 2:**
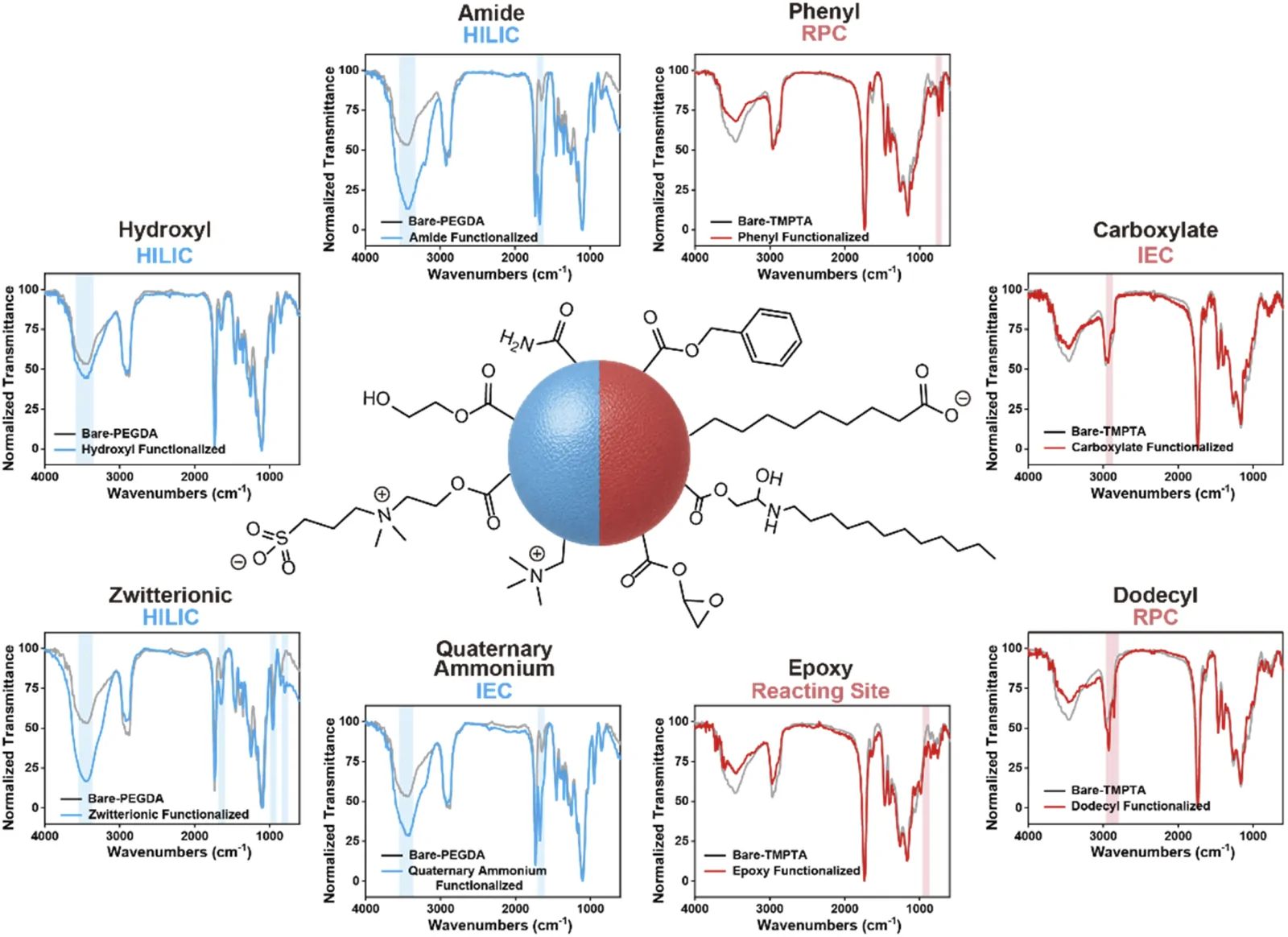
The introduction of versatile functional groups into the hemispheres of the spatially resolved stationary phases, respectively, as characterized by FT-IR spectroscopy. PEGDA: polyethylene glycol diacrylate, TMPTA: trimethylolpropane triacrylate. HILIC: hydrophilic interaction chromatography, RPC: reversed-phase chromatography, IEC: ion-exchange chromatography.

Furthermore, by adjusting the flow rates of the two dispersed phases, the quantitative assembly of stationary-phase chemistry can be realized. Microspheres with quantitative hemisphere ratios from 1 : 5 to 5 : 1 were stereosynthesized at different applied flow rates, among which spatial ratios from 1 : 2 to 2 : 1 are shown in Fig. 3. Optical microscopy characterization proved the stable generation of Janus-structured droplets (Fig. 3b) and the solidification of microspheres with the pre-defined hemisphere ratio (Fig. 3c). The monodispersity and sphericity of the microspheres were characterized by scanning electron microscopy (SEM) (Fig. 3d), indicating their quality as HPLC-grade stationary phase materials, *i.e.* micrometer-scale spherical particles with good monodispersity, which is a prerequisite for high-performance chromatography.^20,21,32^

**Fig. 3:**
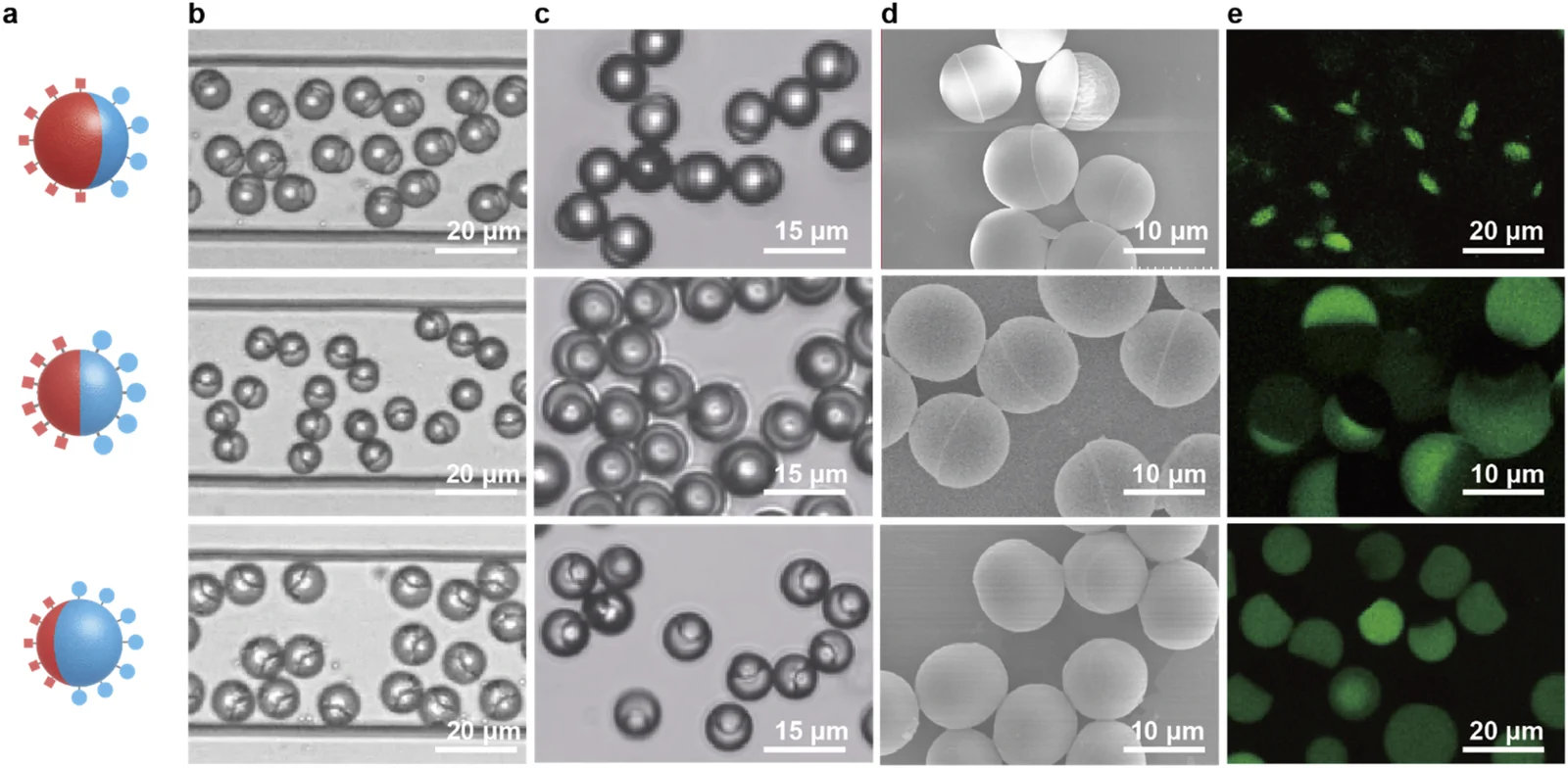
The precision assembly of stationary phases with quantitative ratios. (a) A schematic diagram of spatially resolved microspheres with different hemisphere ratios manufactured by adjusting the flow rates of dispersed phases. (b) Optical microscopy imaging of spatially resolved droplets with different hemisphere ratios. (c) Optical microscopy imaging of spatially resolved microspheres with different hemisphere ratios. (d) SEM imaging of spatially resolved microspheres with different hemisphere ratios. (e) Fluorescence microscopy imaging of spatially resolved microspheres with different hemisphere ratios.

The spatially resolved distribution of functional groups constitutes the basis for the precise definition of chemical selectivity. To characterize the spatial resolution, microspheres with different hemisphere ratios were imaged *via* laser scanning confocal microscopy (LSCM). The fluorescently labelled lipophilic phase (solidified from dispersed phase B) exhibited a distinct hemispherical structure (Fig. 3e), confirming the stereo-architecture of the microspheres. The distribution of functional groups was further investigated by confocal Raman microscopy scanning. As designed, characteristic Raman signals were observed from the phenyl-functionalized hemisphere (Fig. S5), indicating the micrometer-precision spatially resolved application of stationary phase chemistry on the stereosynthesized chromatographic spheres.

### Tuning of separation selectivity

To evaluate the separation performance of the stereosynthesized stationary phases, a series of microspheres with different hemisphere ratios was prepared and packed into chromatographic columns for evaluation.^33–35^ During the random packing and following separation processes, the Janus microspheres did not fracture after exposure to pressure of up to 40 MPa (Fig. S6), demonstrating that the Janus spheres possess sufficient mechanical strength for chromatographic applications.

Spatially resolved stationary phases, quantitatively combining amide (HILIC-type ligand) and C12 (RPC ligand) functionalities, were designed, stereosynthesized and evaluated (Fig. 4). In HILIC mode, the hydrophilic retentivity of cytochrome C was found to increase continuously with an increase in the hydrophilic amide phase content, from 0–100% (Fig. 4a); whereas in RPC mode, hydrophobic analytes, represented by toluene, exhibited an opposite trend as the C12 phase content increased from 0–100% (Fig. 4b). A quantitative evaluation using a series of analytes was further conducted (Fig. 4c). The retention factors (*k*) of both hydrophilic and hydrophobic molecules exhibited a linear relationship with the hemisphere ratios, demonstrating the precise response of retentivity as a function of the applied stationary phase chemistry domain. This indicates that the spatial distribution of the stationary phase, as an unexplored structural parameter, can be used as an independent characteristic for the tuning of chromatographic thermodynamics.

**Fig. 4:**
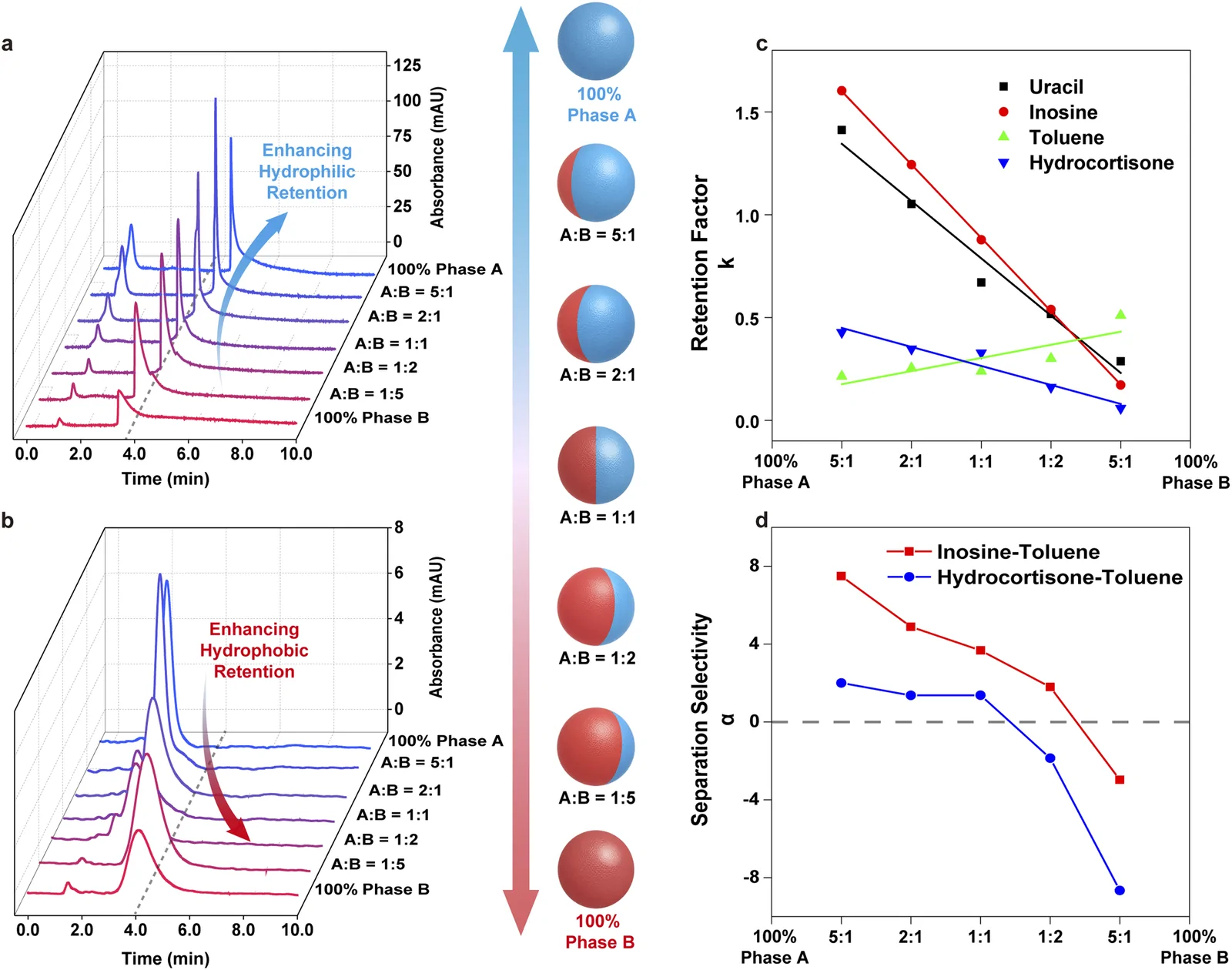
Tuning of the chromatographic thermodynamic properties based on the precision assembly of stationary phase chemistry. (a) The hydrophilic retention of cytochrome C on stationary phases with different hemisphere ratios in HILIC mode; mobile phase: 90–40% ACN in 5 min. (b) The hydrophobic retention of toluene on stationary phases with different hemisphere ratios in RPC mode; mobile phase: 60% ACN. (c) Retention factors of a series of molecular probes as a function of the stationary phase ratio; mobile phase: 60% ACN. (d) Selectivity factors of molecular pairs as a function of the stationary phase ratio; mobile phase: 60% ACN. Phase A: amide phase; phase B: dodecyl phase.

As a result, the different slopes of the retention factor as a function of the stationary phase ratio can be used for tuning the selectivity factor (*α*). With the analyte pairs of inosine and toluene, and hydrocortisone and toluene as examples, respectively, selectivity factors of up to 8 can be realized (Fig. 4d). What is more, due to the widely adjustable separation selectivity factor (ranging from −9 to 8), a complete reversal of selectivity (*i.e.* a reversed elution order) can be achieved with the spatially resolved stationary phase, supporting excellent freedom in terms of selectivity tuning and resolution optimization.

### Rational design of the stationary phase

In conventional stationary phases, functional groups are randomly co-located in the same space, leading to an unorderly distribution of interaction sites. Such co-location of different types of ligands inevitably leads to steric effects, electrostatic repulsion or other types of interference between neighbouring functional groups, making the resultant retention behavior and selectivity unpredictable, which is passively accepted in practical chromatography. In contrast, with stereosynthesized stationary phases, the different functional groups are decoupled both spatially and chemically. This enables one functional group to interact independently with an analyte molecule without the chemical interference from a second functional group seen with classical mixed-mode chromatography materials.

As the two most extensively used HPLC modes, reversed-phase chromatography and hydrophilic interaction chromatography (RPC-HILIC) were used to investigate model stationary phases. Spatially resolved and classical non-spatially resolved stationary phases were prepared (Fig. 5a), both consisting of amide- and C12-functional phases in a 1 : 1 (v/v) ratio, with or without the spatially resolved distribution of functional groups. As shown in Fig. 5b, under low ACN% conditions, the hydrophobic probe toluene exhibited RPC retention on both microspheres. Stunningly, however, 152% retentivity enhancement was observed on the spatially resolved microspheres. Similarly, under high ACN% conditions, the hydrophilic probe uracil showed HILIC retention on both phases, but a significant 213% hydrophilic retentivity enhancement was achieved on the spatially resolved stationary phase. These results highlight that, in comparison with a conventional non-spatially resolved stationary phase, a spatially resolved distribution enables efficient interactions and well-defined retentivity both hydrophobically and hydrophilically. It needs to be pointed out that, in fact, chemically the same amounts of hydrophobic and hydrophilic groups were applied during the synthesis of both spatially resolved and non-spatially resolved spheres; however, as shown in Fig. 5b, significant 150–200% enhancements in hydrophobic and hydrophilic retentivity have been generated on the spatially resolved stationary phase. This is a clear sign that the stereo-precise application of chemistry has released retentivity that originally existed in the non-spatially resolved mixed-mode phase, but that was largely diminished due to the random co-location of functional groups. According to chromatographic theory, for RPC and HILIC modes, it is generally accepted that a partitioning process dominates molecular retention.^36,37^ Rather than being based on one-to-one interactions between analyte molecules and bonded groups, a solvation layer forms on the surface of the stationary phase, and retention arises from molecule partitioning between the solvation layer and the mobile phase.^4–6^ The differential partitioning of molecules leads to the selectivity between them and, therefore, resolution. In microspheres with co-locating amide and C12 groups, the surface hydrophilicity and hydrophobicity are inevitably interrupted, weakening the formation of a stable solvation layer and therefore diminishing the designed retentivity. In contrast, the spatially resolved distribution of stationary phase chemistry enables the effective formation of solvation layers, leading to the simultaneous enhancement of both RPC and HILIC retentivities.

**Fig. 5:**
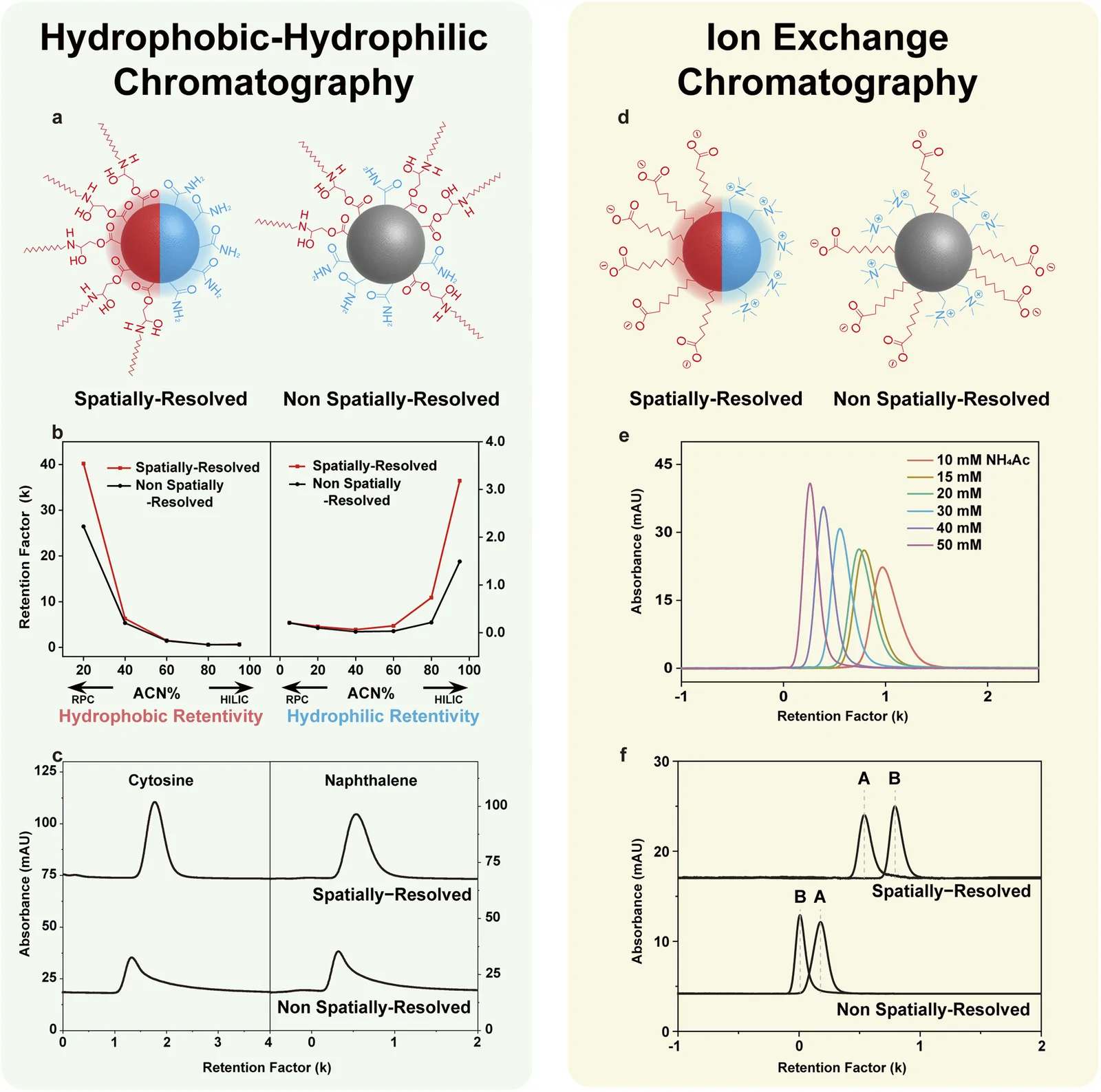
The chromatographic behavior of spatially resolved and non-spatially resolved stationary phases. (a and d) Schematic diagrams of spatially resolved and classical non-spatially resolved mixed-mode stationary phase chemistry for hydrophobic–hydrophilic chromatography and ion-exchange chromatography. (b) The hydrophobic and hydrophilic retentivity of spatially resolved and non-spatially resolved microspheres; probes: toluene for hydrophobic retention, uracil for hydrophilic retention. (c) Chromatography of naphthalene and cytosine on spatially resolved and non-spatially resolved microspheres; mobile phase: 80% ACN. (e) The retention of AMP (adenosine 5′-monophosphate) as a function of the ionic strength of the mobile phase: 10–50 mM NH_4_Ac. (f) Chromatography of benzoic acid (A) and AMP (B) on spatially resolved and non-spatially resolved microspheres; mobile phase: 10 mM NH_4_Ac.

Furthermore, it was observed that probe molecules exhibited greater tailing (asymmetry factor, *A*_s_, = 4.57 for naphthalene and 5.03 for cytosine) on the non-spatially resolved microspheres (Fig. 5c). This suggests that retention on the co-locating phase involves a multiplex interaction process rather than a single dominant mechanism, therefore showing significant secondary interaction effects.^38^ In contrast, the spatially resolved microspheres exhibit distinct retention due to the spatially isolated distribution of the different functional groups, resulting in improved peak shapes (*A*_s_ = 1.54 for naphthalene and 1.28 for cytosine). These findings further highlight the role of the spatially resolved arrangement of stationary phase chemistry in determining retention behavior.

To further demonstrate the significance of spatial distribution in the rational design of stationary phases, ion-exchange interactions were also investigated for mixed-mode chromatography (Fig. 5d). The spatially resolved microspheres consisted of anion-exchange (trimethylammonium) and cation-exchange (C11 carboxylic acid) groups, which are typically used in commercial IEC columns, individually distributed on the two hemispheres, whereas the same groups were randomly co-located on the non-spatially resolved stationary phase. The two IEC mixed-mode stationary phases were prepared, respectively, and their morphologies and chemical compositions (1 : 1) were confirmed (Fig. S7).

To verify the ion-exchange mechanism, the ionic strength of the mobile phase was investigated on the spatially resolved microspheres (Fig. 5e), and a linear relationship was observed between log *k* (retention factor) and log *C* (buffer salt concentration) (Fig. S8), consistent with the characteristic behavior of ion-exchange retention.^4,5^ In a comparison study, as shown in Fig. 5f, the retention of anionic molecules, benzoic acid (A) and AMP (B), was pronouncedly higher on the spatially resolved stationary phase, in contrast to the non-spatially resolved stationary phase, which exhibited negligible retention. Additionally, a reversed elution order of the two anions was also observed on the spatially resolved stationary phase, while almost no difference was noted for cation retention between the two phases (Table S1). The results revealed that in non-spatially resolved microspheres with randomly distributed stationary phases, short-chain ligands (anion-exchanger, trimethylammonium) have their interactions hindered due to both steric effects and electrostatic repulsion from outer long-chain cation exchange ligands (C11 carboxylic acid), leading to weakened chromatographic retention of anionic molecules; whereas on the spatially resolved stationary phase, the unshielded short-chain anion-exchange groups effectively retained the two anions, with higher retention observed for AMP due to its more negative charge state at pH 7.

Regarding the chromatographic kinetics aspect, with 10 µm spatially resolved and non-spatially resolved stationary phases as examples, similar efficiencies from 1000–8000 plates per m have been observed in RPC-HILIC and IEC modes with a series of probe molecules (Table S2).

The above discussion suggests that the spatial distribution of functional groups should be taken into account when designing multi-interaction stationary phases, in addition to general considerations such as ligand species, composition and abundance.^36,39^ In particular, when the stationary phase simultaneously contains hydrophilic and hydrophobic groups, oppositely charged species, or ligands with steric hindrance, randomly distributed chemical functionality may introduce local interference, thereby complicating the retention behavior and leaving it almost impossible to obtain the designed separation selectivity.

## Conclusions

In summary, as an unexplored structural parameter, the spatial distribution of stationary phase chemistry has been introduced as an independent chromatographic feature, and its role in regulating separation thermodynamics has been proven to be designable and tunable. To reach this understanding and show manufacturing capabilities, stereosynthesis at the micrometer scale was developed in order to isolate neighbouring interaction sites and reduce the chemical interference that exists in classical separation materials. The spatially resolved stationary phase thus synthesized presented improved peak shapes, enhanced retention power, and flexible assemblies of functionality, supporting the predefined chemical selectivity. More importantly, stereosynthesis provides a new methodology for the rational design and precision manufacture of separation materials, paving the way towards performance-by-design.

## Author contributions

X. H., K. S., C. H. and Z. Z. contributed equally to this work. X. H., K. S., H. C., Z. Z. and B. Z. conceived the study and designed the research. X. H. and K. S. developed the methodology, performed the main experiments, curated the data, validated the results and carried out the formal analysis. J. C., Q. C. and X. W. contributed to the experimental investigations. H. C. contributed to conceptual development, methodology design, data curation and formal analysis. Z. Z. contributed to conceptual development and methodology design. X. H. and K. S. wrote the original draft. H. C., Z. Z. and B. Z. reviewed and edited the manuscript. Y. S., T. H., N. F. and B. Z. provided resources, supervision and project administration. B. Z. acquired funding and supervised the overall project.

## Conflicts of interest

There are no conflicts to declare.

## Supplementary Material

SC-OLF-D6SC05966J-s001

## Data Availability

All data supporting the findings of this study are included within the article and its supplementary information (SI). Supplementary information: detailed experimental materials, including all reagents and chemicals used in this study and detailed parameters for all experiments. Ref. 40 and 41 are cited in the SI. See DOI: https://doi.org/10.1039/d6sc05966j.
