# Supplementary material for: Stereosynthesis of stationary phase with precision defined separation selectivity
Source: Chem Sci. 2026 Sep 24;17(40):19171–9. doi: 10.1039/d6sc05966j (PMC13631962; doi:10.1039/d6sc05966j)
Supplement: Supplementary file 1 [file SC-017-D6SC05966J-s001.pdf]

Supplementary Information (SI) for Chemical Science.
This journal is © The Royal Society of Chemistry 2026

Supporting Information

Stereosynthesis of Stationary Phase with Precision Defined Separation Selectivity

Xiangyu Huang,[†a] Kaiyue Sun,[†ab] Hanchen Cao,[†a] Zhuoheng Zhou,[†c] Jikai Chen,[a] Qingshu Cai,[a] Xiaofei Wang,[d] Yanling Song,[a] Teng-Xiang Huang,[ae] Ning Fang,[ae] and Bo Zhang*[a]

[a] Department of Chemistry and the MOE Key Laboratory of Spectrochemical Analysis & Instrumentation, College of Chemistry and Chemical Engineering, State Key Laboratory of Vaccines for Infectious Diseases, Xiang An Biomedicine Laboratory, Xiamen University, Xiamen 361005, China

[b] Beijing Institute of Smart Energy, Beijing 102211, China.

[c] Bioanalytical Services, Department of Toxicology, WuXi AppTec (Suzhou) Co. Ltd, Suzhou, China.

[d] ColumnScientific Inc., Xiamen 361102, China.

[e] Pen-Tung Sah Institute of Micro-Nano Science and Technology, Xiamen University, Xiamen 361005, China.

† Xiangyu Huang, Kaiyue Sun, Hanchen Cao and Zhuoheng Zhou contributed equally to this work.

Table of Contents:

Experimental Details

Figure S1. Optimization of the mixing ratio of hydrofluoroether and fluorocarbon.

Figure S2. Schematic illustration of synthesis of fluorosurfactants.

Figure S3. FT-IR spectroscopy of PFPE-PEG-PFPE and PFPT-PPG-PFPE.

Figure S4. Preparation of spatially-resolved droplets/microspheres in a 4-channel microfluidic chip.

Figure S5. Spatially-resolved distribution of functional groups on stereosynthesized microspheres.

Figure S6. Optical microscopic imaging and SEM imaging of the spatially-resolved stationary phase microspheres under 40 MPa in 60% ACN for 6 h.

Figure S7. Morphology and chemical composition of spatially-resolved and non-spatially resolved microspheres.

Figure S8. Characterization of ion exchange interaction on spatially-resolved microspheres under IEC mode.

Table S1. Retention of anions and cations on spatially-resolved and non-spatially resolved mixed-mode IEC stationary phases.

Table.S2. The column efficiencies of five probes on non spatially-resolved and spatially-resolved stationary phases.

References

Experimental Details

Reagents and materials

Silicon Wafers, SU-8 2025 photoresist, SYLGARD 184 silicone elastomer (PDMS prepolymer) were purchased from Cchip Scientific Instrument Co. Ltd. (Suzhou China). Jeffamine D-400, perfluoropolyether carboxylic acid (PFPE-COOH, Mn=7000), Jeffamine ED-900 were purchased from Quzhou Chemwell Chemical Industry Co., Ltd. (Suzhou China). FC-40, HFE-7100, HFE-7500 were purchased from 3M China Ltd. (Shanghai China). Dimethyl carbonate (DMC), polyethylene glycol diacrylate (PEGDA, Mn=600), trimethylolpropane triacrylate (TMPTA), lithium phenyl(2,4,6-trimethylbenzoyl)phosphinate (LAP), diphenyl(2,4,6-trimethylbenzoyl)phosphine oxide (TPO), acryloyl chloride, acrylamide, 3-((2-(methacryloyloxy)ethyl)dimethylammonio)propane-1-sulfonate (SBMA), 2-hydroxyethyl methacrylate (HEMA), allyltrimethylammonium chloride, benzyl methacrylate, 10-undecenoic acid, glycidyl methacrylate (GMA), dodecylamine, trifluoroacetic acid (TFA), anhydrous dichloromethane (DCM), sulfuric acid, hydrogen peroxide, thionyl chloride, triethylamine (TEA), anhydrous ethanol, chloroform, isopropanol (IPA), dimethyl sulfoxide (DMSO), propylene glycol monomethyl ether acetate (PGMEA), trichloro(1H,1H,2H,2H-perfluorooctyl)silane, fluorescein, methanol, acetonitrile were purchased from Shanghai Aladdin Biochemical Technology Co., Ltd. (Shanghai China). All chromatographic-grade standard analytes, including benzoic acid, ammonium acetate, thiourea, toluene, uracil, cytosine, naphthalene, hydrocortisone, amitriptyline, cytochrome C, adenosine 5'-monophosphate (AMP) were purchased from Shanghai Aladdin Biochemical Technology Co., Ltd. (Shanghai China)

Design and fabrication of microfluidic chip

To prepare Janus droplets and microspheres, a droplet microfluidic chip with a flow-focusing structure was designed (Figure 1a). The chip consists of four parallel droplet-generating structures, in each of which two dispersed phase channels converge at the nozzle and are subsequently cut into Janus droplets by continuous phase. The microfluidic chip was fabricated by a classical PDMS soft lithography process. Briefly, the CAD design was transferred into a transparency film or chromium plate as a lithography mask (Qingyi Photomask Ltd. Shenzhen China). A silicon wafer spin coated by SU-8 photoresist was soft-baked at 65℃ and 95℃, and was exposed by UV under the lithography mask. After post-bake, development, hard-bake and fluorinated, the SU-8 pattern was obtained. PDMS prepolymer A and B were mixed at a ratio of 10:1 (w/w). After degassing, prepolymer was poured onto the SU-8 pattern and placed at 80°C for 4 h to complete curing. The PDMS pattern was carefully cut using a scalpel, and bonded to glass under O_2 plasma treatment. The microfluidic channel was treated by 1.0 wt% trichloro(1H,1H,2H,2H-perfluorooctyl)silane (dissolved in FC-40) prior to use and drying at 100 °C to remove the solvent.

Synthesis of fluorosurfactant

Since fluorinated oil was chosen as the continuous phase, fluorosurfactant was added to stabilize the droplets, which was synthesized according to previous literature with

modifications.[40,41] Specifically, 3 g of perfluoropolyether carboxylic acid (PFPE-COOH, Mn=7000) was dissolved in 25 mL of HFE-7100. 312 μL of $SOCl_2$ was added under magnetic stirring and N_2 atmosphere, and the reaction was refluxed at 65 °C for 24 h to obtain PFPE-COCl. Subsequently, solvent and excess $SOCl_2$ was removed in vacuum, and PFPE-COCl was re-dissolved by 17 mL HFE-7100 and 17 mL CH_2Cl_2. 0.53 e.q. Jeffamine ED-900 or polyetheramine D-400 was dissolved in 17 mL CH_2Cl_2 and 90 μL triethylamine was added as acid binding agent. After addition of PFPE-COCl, the reaction was refluxed at 65°C for 24 h under N_2 atmosphere. The solvent was removed in vacuum to obtain the surfactant. The prepared surfactant was re-dissolved with 5 mL HFE-7100, washed sequentially with 40 mL methanol and CH_2Cl_2, and centrifuged at 5000 rpm for 5 min. After dried under vacuum at 50 °C, a white oil was obtained.

The synthesized fluorosurfactants were characterized by IR spectroscopy. Compared to PFPE-COOH, the absorption peak of C=O stretching vibration of $PFPE_2$-Jeffamine and $PFPE_2$-Polyetheramine were blue shifted, and C-H absorption peaks appeared, which proved the successful synthesis of the target surfactants (Figure S3).

Preparation of spatially-resolved stationary phase.

The preparation of spatially-resolved stationary phase was performed on the droplet microfluidic chip described above. The continuous phase was 3:1 (v/v) HFE-7500/FC-40 with 2 wt% of fluorosurfactant $PFPE_2$-Jeffamine or $PFPE_2$-Polyetheramine. Dispersed phase A consisted of water (50 vol%) and PEGDA-400 (50 vol%) with the addition of water-soluble initiator LAP at a concentration of 2.5 mg/mL. Disperse phase B consisted of DMC (50 vol%) and TPTA (50 vol%), and initiator TPO was added at a concentration of 25 mg/mL.

Dispersed phase and continuous phase were introduced into the chip by a syringe pump with a continuous phase flow rate of 1.0 μL/min and a total dispersed phase flow rate of 0.4 μL/min. The generated droplets were collected using a transparent PTFE tube (0.38 mm i.d.) and then cured by exposure to 360 nm UV light. The cured microspheres were washed by 2:1 (v/v) MeOH/HFE-7100 to remove the surfactant, centrifuged and dispersed in MeOH for further use.

Chromatography evaluation.

Spatially-resolved stationary phase microspheres were dispersed in DMSO for use in RPC–HILIC mode. For microspheres used in IEC mode, 100 mM NH_4Ac was added to balance the surface charge. The microspheres were then dispersed to form slurries at approximately 20 mg/mL. Slurry was injected into a 150 μm i.d. capillary by a syringe, and a single-particle frit was filled at one end of the capillary.[33-35] After compressed up to a maximum pressure of 2900 psi by a high-pressure pump with methanol as the packing and washing solvent, the column of target length was cut off by a ceramic piece, and another single-particle frit is filled at the other end of the column.

Columns packed with spatially-resolved stationary phase were characterized on a nano-HPLC system (U-3000nano, ThermoFisher Scientific). The flow rate was set at 750 nL/min. For

separations in RPC-HILIC mode, mobile phase A is H_2O, and mobile phase B is ACN. For separations in IEC mode, mobile is 50% ACN containing NH_4Ac at concentrations of 10, 15, 20, 30, 40, or 50 mM. Sample was dissolved in a solvent with the same composition as the mobile phase and at a concentration of 2 mg/mL. The injection volume was 4 nL, and detection was performed at UV 214 nm or 254 nm.

The selectivity factor (α) was calculated as $\alpha = k_2 / k_1$, where k_1 and k_2 are the retention factors (k) of the two analytes investigated, and k_2 corresponds to the later-eluting one. When reversal of the elution order occurred, α was reported as a negative value.[4,5]

Supporting Figures

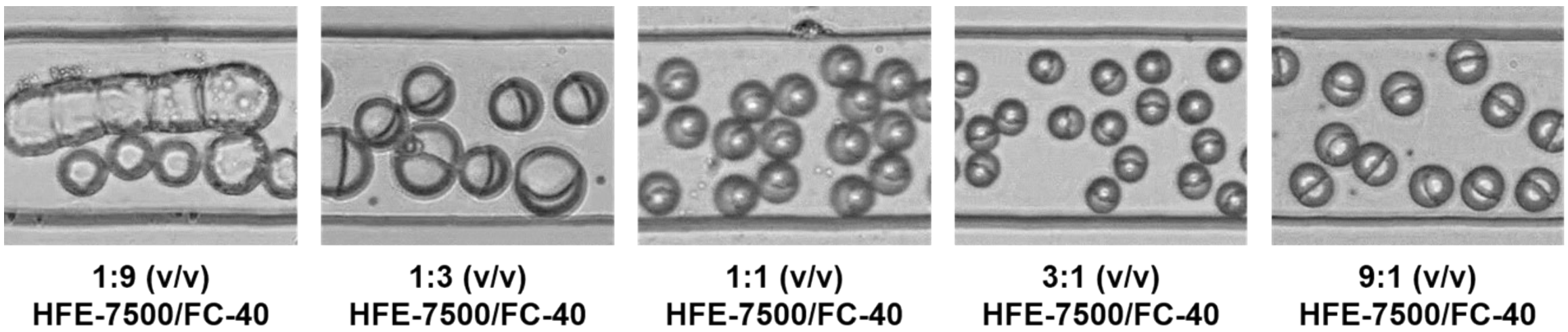

Figure S1. Optimization of the mixing ratio of hydrofluoroether and fluorocarbon.

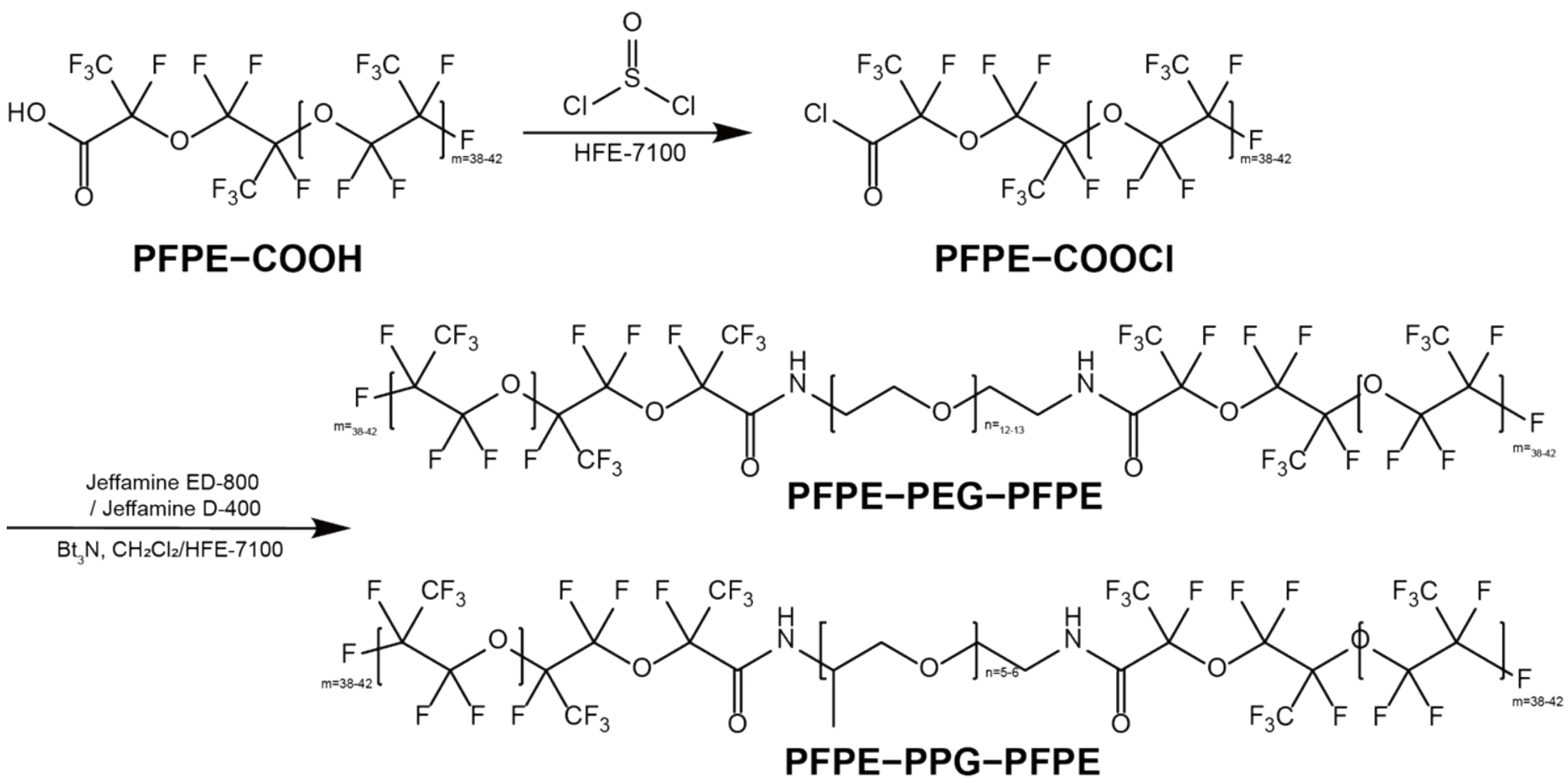

Figure S2. Schematic illustration of synthesis of fluorosurfactants.

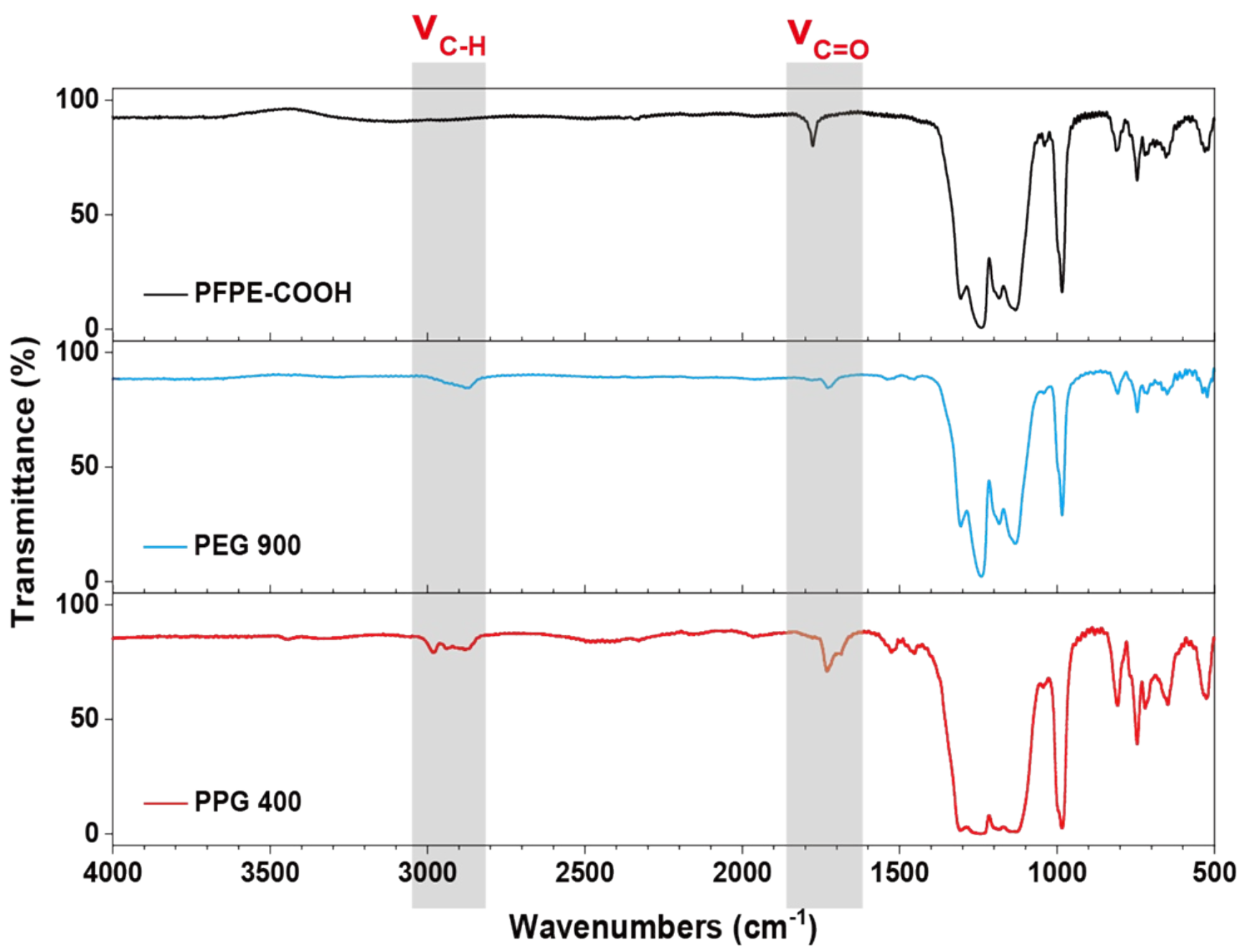

Figure S3. FT-IR spectroscopy of PFPE-PEG-PFPE and PFPT-PPG-PFPE. Inset of panel highlights the shift of the C=O and C-H stretch during synthesis of the fluorosurfactants.

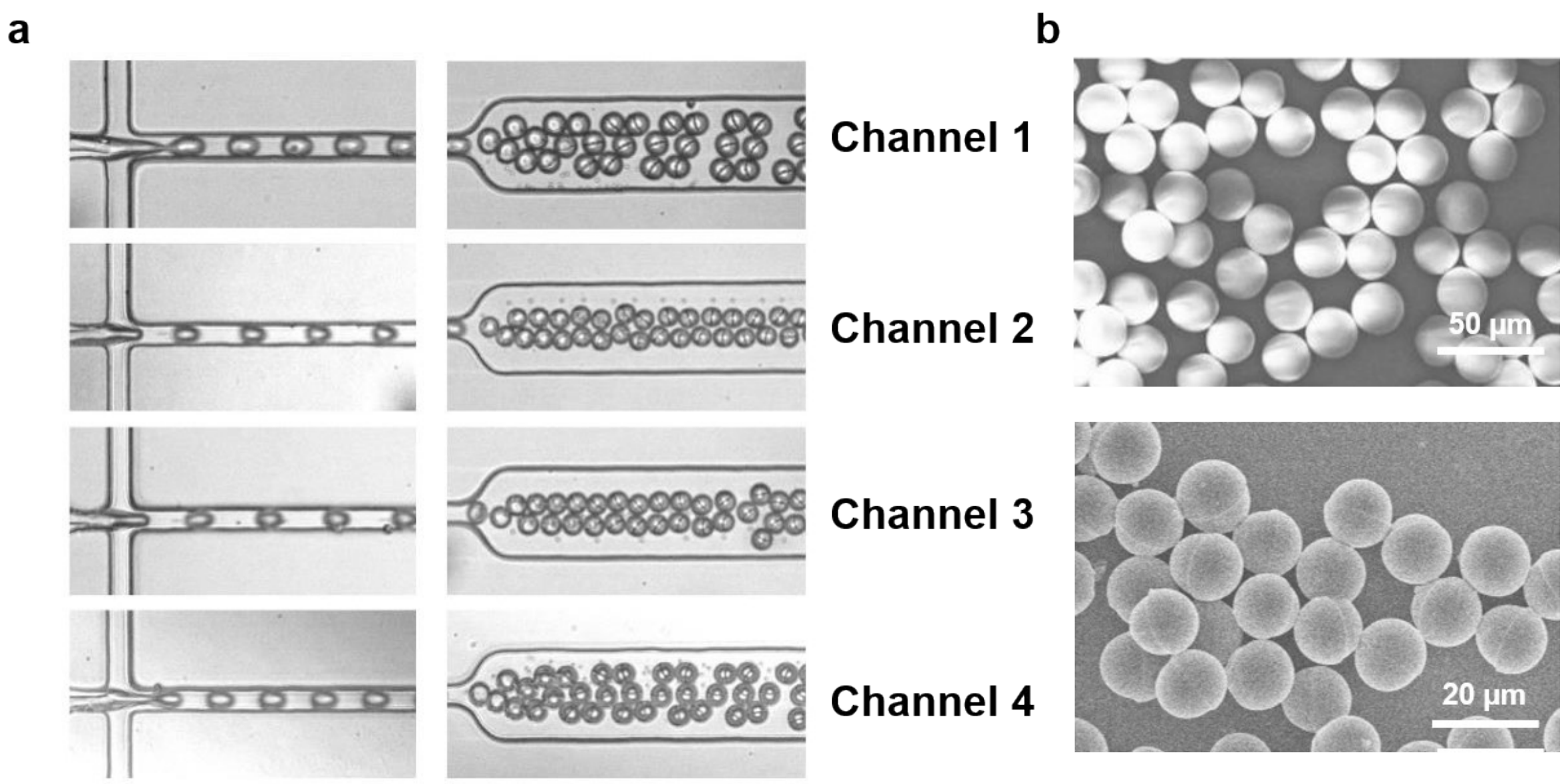

Figure S4. Preparation of spatially-resolved droplets/microspheres in a 4-channel microfluidic chip. a) Optical microscopic imaging of Janus-structured droplets under generation. b) Monodispersity of Janus-structured microspheres.

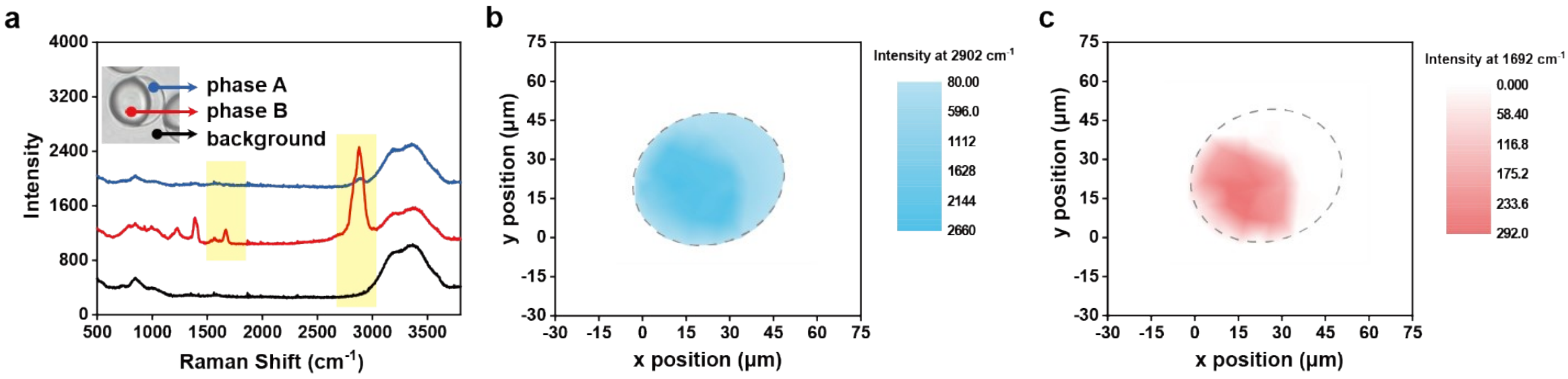

Figure S5. Spatially-resolved distribution of functional groups on stereosynthesized microspheres. a) Raman spectroscopy of hemisphere A (phenyl functioned), hemisphere B (non-phenyl functioned) and the background (aqueous solution) of phenyl-functional Janus-structured microsphere. Inset of panel highlights the characteristic signal differences between the two hemispheres and between the microsphere and the background. b) Confocal Raman discrimination with the microsphere and between the microsphere and the background. Intensity at 2902 cm^{-1}. c) Confocal Raman discrimination between hemisphere A and hemisphere B. Intensity at 1692 cm^{-1}.

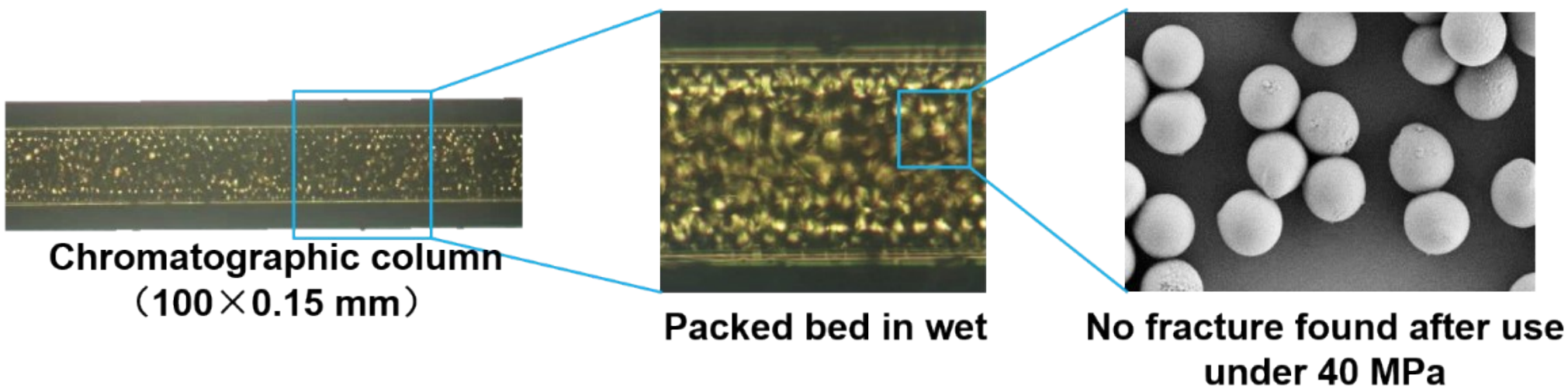

Figure S6. Optical microscopic imaging and SEM imaging of the spatially-resolved stationary phase microspheres under 40 MPa in 60% ACN for 6 h.

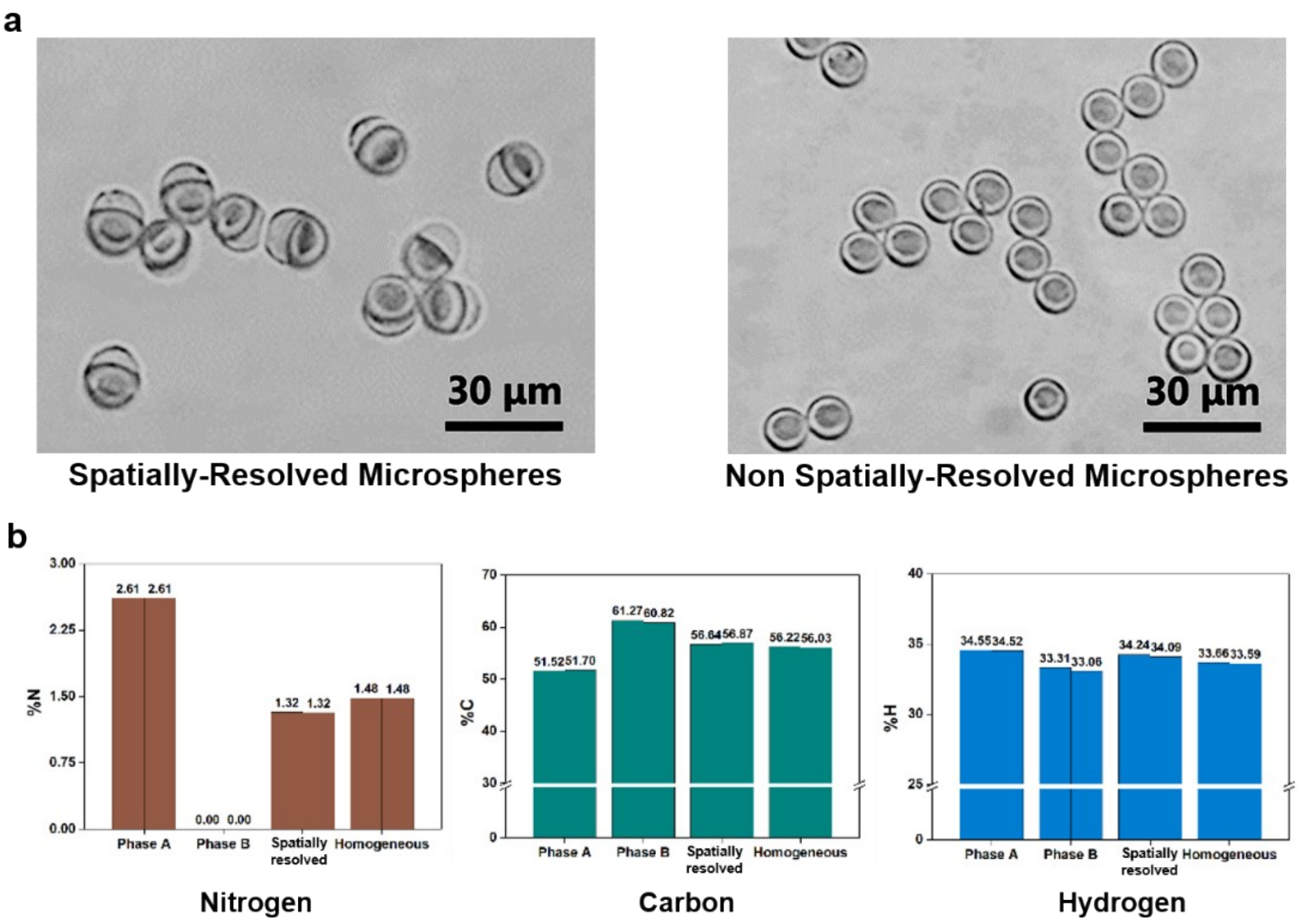

Figure S7. Morphology and chemical composition of spatially-resolved and non-spatially resolved microspheres. a) Optical microscopic imaging of monodisperse spatially-resolved and non-spatially resolved microspheres. b) Elemental contents of spatially-resolved and non-spatially resolved microspheres. Phase A: trimethylammonium functionalized phase; Phase B: carboxyl functionalized phase.

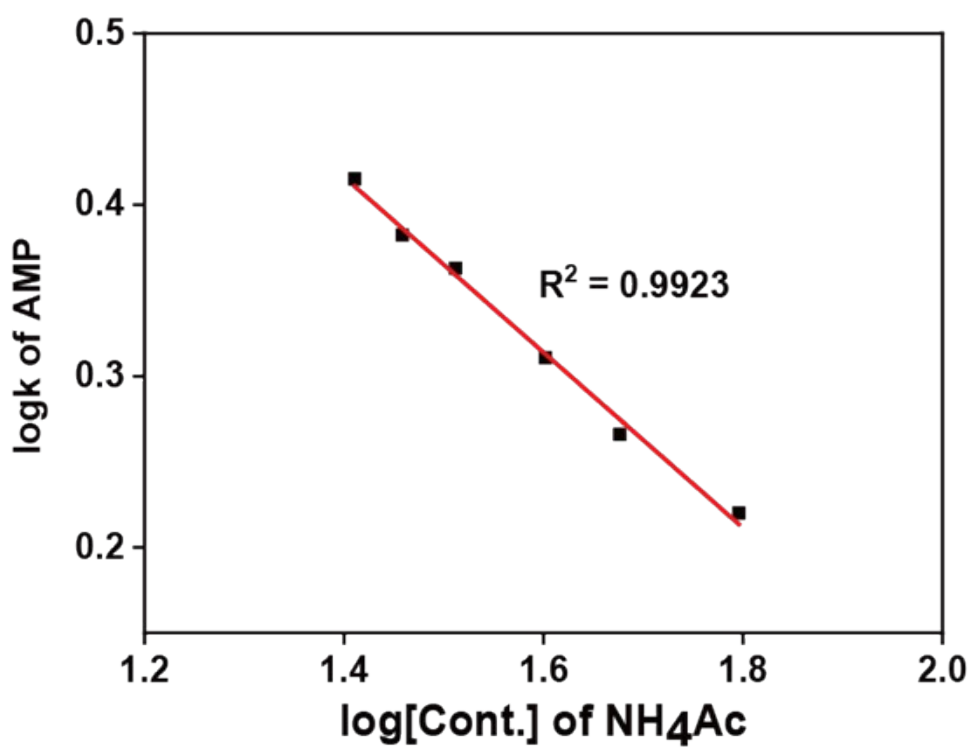

Figure S8. Characterization of ion exchange interaction on spatially-resolved microspheres under IEC mode. Correlation between retention factor (log *k*) and buffering salt concentration (log *C*).

Supporting Tables

Table S1. Retention of anions and cations on spatially-resolved and non-spatially resolved mixed-mode IEC stationary phases.

Type	Analyte	Stationary phase	Retention Factor (*k*)
Anion	AMP	Spatially-resolved	0.79
		Non-spatially resolved	0.01
	Benzoic Acid	Spatially-resolved	0.54
		Non-spatially resolved	0.18
Cation	Amitriptyline	Spatially-resolved	6.00
		Non-spatially resolved	6.15

Table.S2 The column efficiencies of five probes on non spatially-resolved and spatially-resolved stationary phases.

Analyte	Column efficiency (plates per meter)	
	Non spatially-resolved*	Spatially-resolved*
Naphthalene	4380	3200
Cytosine	2700	3530
AMP	1160	3230
Benzoic Acid	1080	1030
Amitriptyline	7290	8740

* Both are 10 μm spherical particles packed into 150 μm i.d. x 100 mm length columns

Reference

4 L. R. Snyder, J. J. Kirkland, and J. W. Dolan, Introduction to Modern Liquid Chromatography, 3rd ed.; Wiley: Hoboken, NJ, 2010.

5 C. F. Poole, The Column in Liquid Chromatography. The Essence of Chromatography; Elsevier: Amsterdam, 2003.

33 X. Wang, J. Zhu, C. Yang, F. Qin, and B. Zhang, Segmented Microfluidics-Based Packing Technology for Chromatographic Columns. *Anal. Chem.* 2021, **93** (24), 8450–8458. DOI: 10.1021/acs.analchem.1c00545

34 Y. Liu, H. Wen, S. Chen, X. Wang, X. Zhu, L. Luo, X. Wang, and B. Zhang, Mass Fabrication of Capillary Columns Based on Centrifugal Packing. *Anal. Chem.* 2022, **94** (22), 8126–8131. DOI: 10.1021/acs.analchem.2c00442

35 Z. Xiao, L. Wang, Y. Liu, Q. Wang, and B. Zhang, A "Plug-and-Use" Approach towards Facile Fabrication of Capillary Columns for High Performance Nanoflow Liquid Chromatography. *J. Chromatogr. A* 2014, **1325**, 109–114. DOI: 10.1016/j.chroma.2013.12.002

40 C. Holtze, A. C. Rowat, J. J. Agresti, J. B. Hutchison, F. E. Angilè, C. H. J. Schmitz, S. Köster, H. Duan, K. J. Humphry, R. A. Scanga, J. S. Johnson, D. Pisignano, and D. A. Weitz, Biocompatible Surfactants for Water-in-Fluorocarbon Emulsions. *Lab Chip* 2008, **8** (10), 1632–1639. DOI: 10.1039/B806706F

41 W. L. Matochko, S. Ng, M. R. Jafari, J. Romaniuk, S. K. Y. Tang, and R. Derda, Uniform Amplification of Phage Display Libraries in Monodisperse Emulsions. *Methods* 2012, **58** (1), 18–27. DOI: 10.1016/j.ymeth.2012.07.012
